# The role of long noncoding RNAs as regulators of the epithelial–Mesenchymal transition process in oral squamous cell carcinoma cells

**DOI:** 10.3389/fmolb.2022.942636

**Published:** 2022-08-29

**Authors:** Zifei Shao, Xiang Wang, Yiyang Li, Yanjia Hu, Kun Li

**Affiliations:** ^1^ Xiangya School of Stomatology, Central South University, Changsha, Hunan, China; ^2^ Hunan Clinical Research Center of Oral Major Diseases and Oral Health and Xiangya Stomatological Hospital, Changsha, China

**Keywords:** lncRNAs, OSCC, EMT, mechanism, signaling pathways

## Abstract

Oral squamous cell carcinoma (OSCC) is a highly invasive and relatively prevalent cancer, accounting for around 3% of all cancers diagnosed. OSCC is associated with bad outcomes, with only 50% overall survival (OS) after five years. The ability of OSCC to invade local and distant tissues relies on the induction of the epithelial–mesenchymal transition (EMT), wherein epithelial cells shed their polarity and cell-to-cell contacts and acquire mesenchymal characteristics. Consequently, a comprehensive understanding of how tumor cell EMT induction is regulated has the potential of direct attempts to prevent tumor progression and metastasis, resulting in better patient outcomes. Several recent studies have established the significance of particular long noncoding RNAs (lncRNAs) in the context of EMT induction. Moreover, lncRNAs regulate a vast array of oncogenic pathways. With a focus on the mechanisms by which the underlined lncRNAs shape the metastatic process and a discussion of their potential utility as clinical biomarkers or targets for therapeutic intervention in patients with OSCC, the present review thus provides an overview of the EMT-related lncRNAs that are dysregulated in OSCC.

## Introduction

In both developed and developing countries, cancer is by far the leading cause of mortality. Cancer remains a chronic risk to public health worldwide, with an estimated 19.3 million diagnoses and 9.9 million cancer-associated deaths in 2020 alone (Sung et al., 2021). Oral cancer is the 18th most prevalent tumor subtype, with approximately 370,000 patients diagnosed in 2020 (Sung et al., 2021) and over 300,000 new diagnoses annually, together with 145,000 cases of oral cancer-related mortality (Thomson, 2018). Oral squamous cell carcinoma (OSCC) represents more than 90% of all oral tumors (Ali et al., 2017). Mid-southern Asia has extremely high OSCC incidence rates; however, in northern and eastern Europe, an elevated OSCC incidence rate was also recorded (Johnson et al., 2020). The majority of OSCC treatment options involve a combination of surgery, radiotherapy, and chemotherapy. The 5-year OS of affected individuals is ∼50% due to the rapid advancement of the metastatic disease. In this view, the mechanism underlying OSCC tumor metastasis must be elucidated in more detail to improve the prognostic assessment of affected individuals (Chi et al., 2015).

Numerous microarray- and RNA-seq-based studies have revealed the dysregulation of lncRNA expression in various types of carcinomas (Huarte, 2015). Notably, many of these lncRNAs have been shown to play critical regulatory roles in the induction of EMT in tumor cells, thus supporting metastatic growth (Liu et al., 2020). The present review thus provides an overview of the molecular mechanisms linking lncRNAs and EMT induction in OSCC.

## Role of epithelial–Mesenchymal transition induction in tumor invasion and metastasis

**FIGURE 1:**
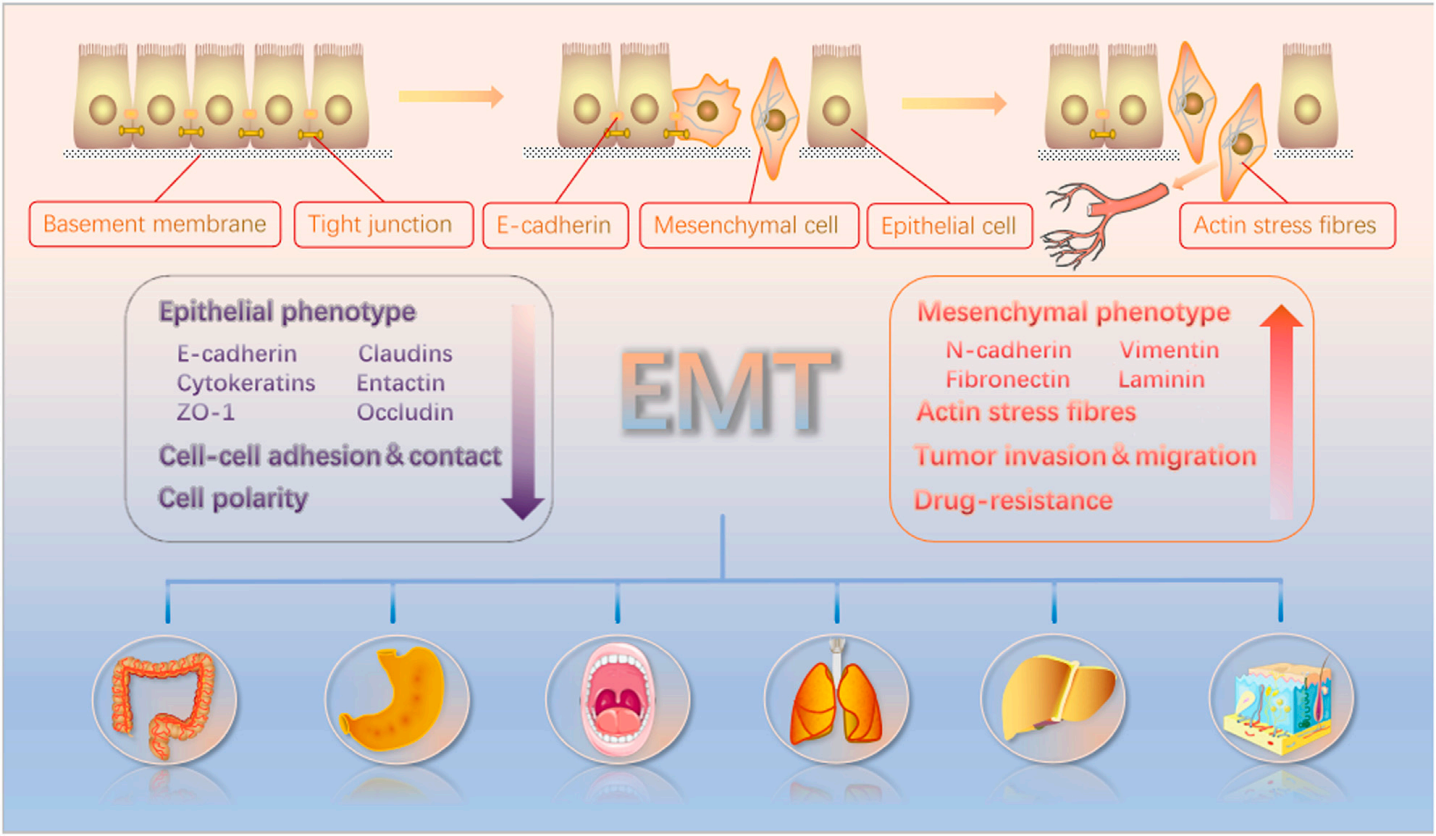
EMT causes polarized epithelial cells to acquire characteristics similar to motile mesenchymal cells. These cells can then detach from the basement membrane and disseminate from the whole body through the bloodstream. The EMT induction results in enhanced tumor cell invasion, migration, and resistance to drug treatment. Furthermore, epithelial and mesenchymal biomarkers and their changes in the context of EMT induction are noted in the figure.

EMT is a complex process by which the epithelial-derived cells lose their polarity and associated features and adopt mesenchymal-like properties. The EMT is broadly classified into three types that are involved in embryogenesis, wound healing/tissue fibrosis, and the malignant progression of epithelial cell-derived tumors (Kalluri and Weinberg, 2009). During embryo development, tight junctions and other structures, including desmosomes, gap junctions, and adheren junctions, link epithelial cells to the basement membrane (Yang and Weinberg, 2008). Eventually, however, these cells lose their polarity and, when developmentally mature, become more motile and invasive (Lim and Thiery, 2012). This same process can also occur in tumor cells of epithelial origin. They detach from the basement membrane and enter the systemic circulation, where they can start metastatic tumor growth in other parts of the body (Figure 1) (Horejs, 2016). This process is characterized by a decrease in the expression levels of marker genes such as E-cadherin. It is transcriptionally controlled by proteins including Zeb, Twist, Snail, and FoxC2. This increases or suppresses the E-cadherin expression while activating various related signaling pathways such as the Notch, Wnt, NF-κB, and TGF-β pathways (Lamouille et al., 2014; Dongre and Weinberg, 2019).

## Function-based significance of lncRNAs as regulators of the epithelia–Mesenchymal transition induction in oral squamous cell carcinoma cells

**FIGURE 2:**
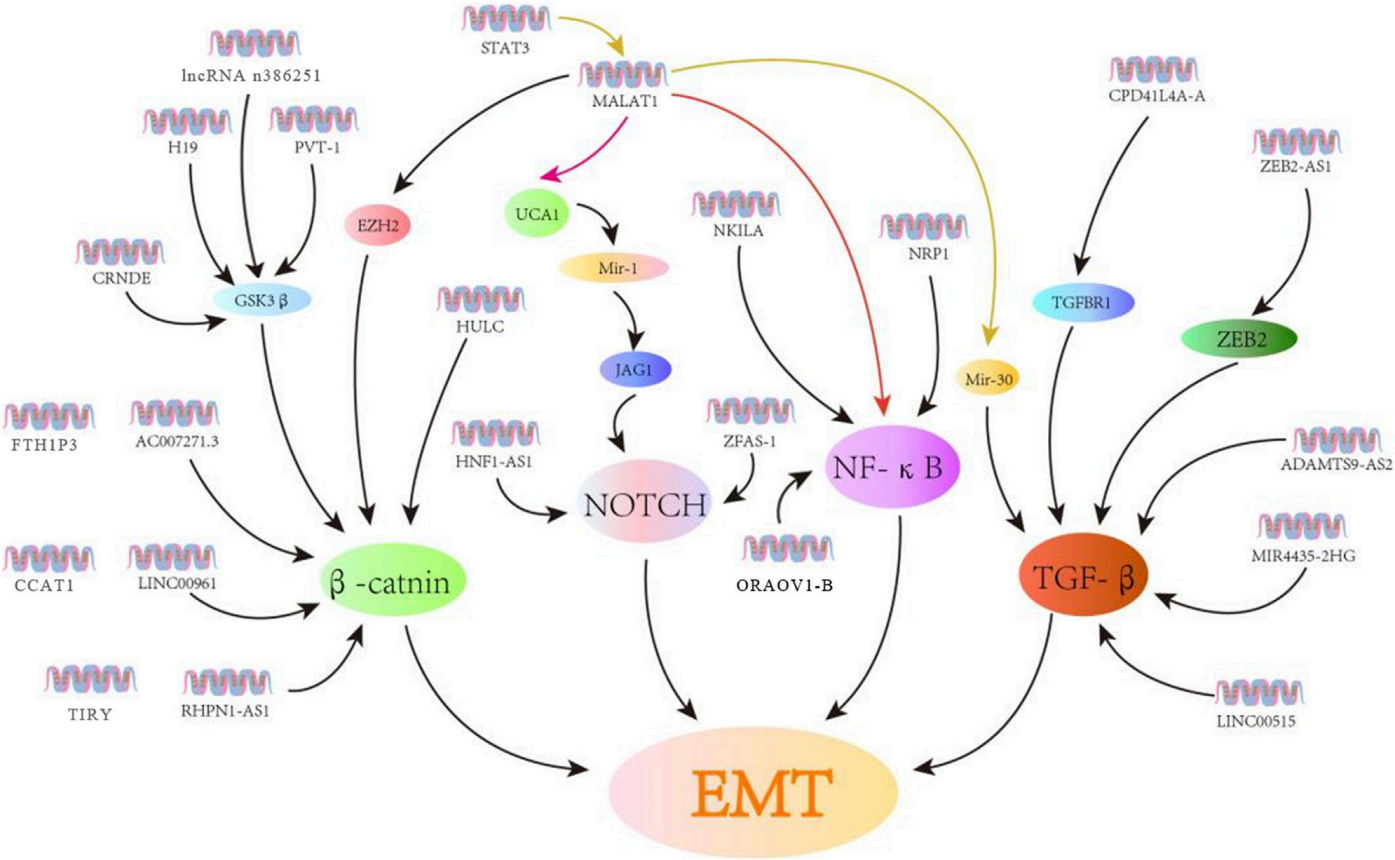
LncRNAs control EMT induction in OSCC cells through various signaling cascades. Herein, the lncRNAs associated with EMT are related to the Wnt, Notch, NF-κB, and TGF-β signaling cascades.

Noncoding RNAs (ncRNAs) are separated into different length-based categories (Esteller, 2011), with short (18–25 nt) microRNAs (miRNAs) being capable of regulating over half of all of the genes expressed in mammals (Simonson and Das, 2015). In contrast, lncRNAs comprised of >200 nucleotides can control biological processes through multiple distinct mechanisms. For instance, some lncRNAs interact with antisense mRNAs or bind to sequester target miRNAs to regulate the post-transcriptional gene regulation. In contrast, others recruit chromatin-modifying proteins or transcription factors to particular regions by binding them, while some engage in *cis* protein interactions to facilitate transcriptional regulation (Engreitz et al., 2016). The underlined mechanisms enable lncRNAs to shape key malignant processes in diseases such as renal, bladder, and prostate cancers (Cheng et al., 2019). The importance of lncRNAs as regulators of OSCC cell’s invasion and metastasis has been a focus of recent interest. Moreover, the EMT induction and associated signaling cascades are necessary for metastatic tumor progression. Specific lncRNAs have the potential to influence OSCC cell EMT induction by interacting with a range of targets in the Wnt, TGF-β, Notch, and NF-κB pathways either directly or indirectly by interacting with the target miRNAs (Figure 2; Table 1).

**TABLE 1:** LncRNAs promote OSCC development via multiple regulatory signaling pathways.

Function	Pathway	Author, publication year	LncRNA	Target	References
Upregulation of N-cadherin, vimentin, and fibronectin; downregulation of E-cadherin	Wnt	Xiao, H., et al., 2015	RHPN1-AS1	β-catenin	Qiu et al. (2019)
		Dai, J., et al., 2019	CRNDE	p-GSK-3β, β-catenin,	Dai et al. (2019)
		Zhang, D.M., et al., 2017	H19	β-catenin, GSK-3β, cyclinD1, c-Myc	Zhang et al. (2017)
		Yu, C., et al., 2018	PVT1	β-catenin, GSK3β	Wang and Zhang (2019)
		Zhang, L., et al., 2019	LINC00961	β-catenin	Jiang et al. (2019)
		Liang, J., et al., 2017	MALAT1	Ezh2, β-catenin	Liang et al. (2017)
		Shao, T.R., et al., 2019	AC007271.3	β-catenin, cyclinD1, c-myc, Bcl-2	Shao et al. (2019)
		Liu, W., et al., 2021	lncRNA n386251	p-GSK-3β	Liu et al. (2021)
		Liu, M., et al., 2018	FTH1P3	β-catenin	Liu et al. (2018a)
		Li, G.H., et al., 2019	CCAT	β-catenin	Li et al. (2019a)
		Jin, N., et al., 2020	TIRY	β-catenin	Jin et al. (2020)
	TGF-β	Diao, P., et al., 2019	ZEB2- AS1	ZEB2	Diao et al. (2019a)
		Li, Y., et al., 2019	ADAMTS9-AS2	MRE	Li et al. (2019b)
		Huang, T., et al., 2018	EPB41L4A-AS2	TGFBR1, SMAD2, SMAD3	Huang et al. (2018a)
		Huang, T., et al., 2018	LINC00515		Huang et al. (2018a)
		Huang, T., et al., 2018	miR4435-2HG		Huang et al. (2018a)
	Notch	Zhang, T.H., et al., 2019	UCA1	JAG1	Zhang et al. (2019b)
		Liu, Z., et al., 2019	HNF1A-AS1	Notch1, Hes1	Liu et al. (2019)
		Gao, K., et al., 2017	ZFAS1	EIF4E, NOTCH1	Gao et al. (2017)
	NF-κB	Huang, W., et al., 2016	NKILA	IκBα, NF-κB	Wu et al. (2018)
		Zhou, X., et al., 2015	Malat1	NF-κB, p65	Zhou et al. (2015)
		Chu, W., et al., 2014	NRP1	IκB, p65	Chu et al. (2014)
		Luo, X., et al., 2021	ORAOV1-B	NF-κB, p65	Luo et al. (2021)

## Significance of lncRNAs as epithelial–Mesenchymal transition induction regulators in oral squamous cell carcinoma cells

### Wnt/β-catenin signaling pathway

The Wnt signaling pathway is a highly conserved regulator of numerous cellular processes. The dysregulation of Wnt contributes to the advancement of a variety of malignancies. For instance, in melanoma, Wnt5a can increase protein kinase C (PKC)-mediated EMT induction, hence facilitating metastasis (Dissanayake et al., 2007). In contrast, WNT974 can interfere with the Wnt signaling and suppress the growth of head and neck squamous cell carcinoma (HNSCC) cells (Giefing et al., 2016). The deregulation of critical Wnt pathway-associated proteins such as catenin β1 (CTNNB1), axis inhibition protein 1 (AXIN1), casein kinase 1α (CSNK1A1), glycogen synthase kinase 3 β (GSK3β), and T-cell factor 4 (TCF4) can eventually affect tumor cell invasion and pathways correlated with malignancies (Qiu et al., 2019).

The lncRNA RHPN1-AS1 is overexpressed in many OSCC cancers, which is associated with histological tumor grade. When RPHN-AS1 is silenced in HNSCC cells, this hampers their ability to grow and participate in invasion and metastasis. This is accompanied by the decrease in the expression of vimentin, β-catenin, and claudin-1 (Qiu et al., 2019). Similarly, the knockdown of the lncRNA CRNDE (colorectal neoplasia differentially expressed) can decrease the N-cadherin, vimentin, Snail, p-GSK-3β, and β-catenin expression (Dai et al., 2019). The β-catenin/GSK3β signaling can be inactivated *via* downregulating the H19 lncRNA in tongue squamous cell carcinoma (TSCC) cells (Zhang et al., 2017) or through overexpressing the PVT1 (plasmacytoma variant translocation 1) lncRNA in OSCC and HNSCC cells (Wang and Zhang, 2019). Moreover, the LINC00961 overexpression can reduce the β-catenin expression, and silencing the underlined lncRNA can increase tumor cell invasion and proliferation *via* the Wnt/β-catenin pathway activation (Zhang et al., 2019a). MALAT1 (metastasis-associated lung adenocarcinoma transcript 1) can activate Wnt/β-catenin signaling and induce the EMT induction in TSCC, with the Wnt pathway activator, that is, EZH2 (enhancer of zeste homolog 2), mediating the underlying mechanisms. Specifically, EZH2 interacts with MALAT1 and thus facilitates the nuclear translocation of β-catenin, promoting the EMT and protecting cells from apoptosis (Liang et al., 2017). The AC007271.3 lncRNA has been correlated with the regulation of Wnt/β-catenin signaling in OSCC cells to prevent apoptosis while augmenting proliferative, invasive, and metastatic activity (Shao et al., 2019). Similar to how the FTH1P3 lncRNA modulates PI3K/Akt/GSK3β/Wnt/β-catenin signaling to promote oncogenesis, LOLA1 (lncRNA oral leukoplakia progressed associated 1) can influence AKT/GSK‐3β signaling to assist the EMT induction (Liu et al., 2018a). The ability of the CCAT1 lncRNA to control OSCC cell proliferative, invasive, and migratory activity is tied to its inhibition of miR-181a and consequent activation of the Wnt/β-catenin pathway activity (Li et al., 2019a). The overexpression of the lncRNA TIRY in cancer-associated fibroblasts (CAFs) can also contribute to OSCC cell metastasis through the Wnt/β-catenin pathway activity mediated by serving as a molecular sponge for miR-14 (Jin et al., 2020).

### TGF-β signaling pathway

TGF-β is a cytokine secreted by cells and controls recipient cells’ proliferative activity, invasion, and differentiation. TGF-β can suppress tumor growth and promote apoptotic death in precancerous cells during early tumorigenesis. However, this suppressive activity is lost during the later stages of tumor growth such that TGF-β can stimulate the EMT induction.

According to the Diao et al. (2019a) reported study, the lncRNA ZEB2-AS1 is an essential mediator of *in vitro* EMT induction in response to TGF-β1, with the silencing of ZEB2-AS1 resulting in the ZEB2 mRNA stabilization that ultimately suppresses the ability of HNSCC cells to proliferate, invade, and metastasize. Consistently, the loss of ZEB2-AS1 expression is associated with reduced N-cadherin expression and increased E-cadherin expression, consistent with the ability of the lncRNA to support the EMT induction within HNSCC cells. The ADAMTS9-AS2 lncRNA can suppress AKT signaling activity and control the expression of associated EMT biomarker genes in a conducive manner to inhibit the progression of precancerous oral submucous fibrosis (OSF) lesion to OSCC onset (Zhou et al., 2021). The STAT3 (signal transducer and activator of transcription 3) transcriptional regulator contributes to the EMT induction in many malignancies (Agarwal et al., 2015). Furthermore, TGF-β can promote STAT3 activation and the corresponding EMT induction in HNSCC cells, and knocking out STAT3 influences the TGF-β/MALAT1/miR-30 regulatory axis (Wang et al., 2018a). Based on the study by Teng et al., the TGF-β pathway-related lncRNAs EPB41L4A-AS2, LINC00515, and miR4435-2HG are dysregulated in the head and neck carcinoma, with EPB41L4A-AS2 serving to repress TGFBR1 and limit TGF-β's ability to drive the EMT progression. However, the regulatory roles of the remaining lncRNAs have not yet been fully explored. The lnRNA PAPAS (promoter and pre-rRNA antisense) can enhance TGFβ1 and thus promote the invasion and metastasis of OSCC. However, this process may or may not be related to the EMT induction (Huang et al., 2018a).

### Notch signaling pathway

The Notch signaling pathway is an evolutionarily conserved correlated pathway with successful intercellular communication and proper tissue, cell, and organ development. It is hypothesized that the lncRNA-mediated modulation of Notch signaling influences crucial oncogenic processes in a variety of tumor types with the dysregulated Notch pathway activation.

According to Zhang et al. (2019b), urothelial cancer associated 1 (UCA1) can influence tongue cancer cell malignancy *via* the miR-124/JAG1 signaling axis. Knocking down UCA1 reduces the TGFβ1‐induced EMT induction and invasion in these cells through miR-124-mediated JAG1 regulation and Notch signaling, whereas the miR-124 knockdown has the opposite effect. The STAT3-induced upregulation of HNF1A antisense RNA 1lncRNA (HNF1A-AS1) can promote the Notch pathway activation and OSCC progression *via* HES-1, with HNF1A-AS1 also offering significance as a prognostic biomarker in patients with this carcinoma (Liu et al., 2019). Knocking down the ZNFX1 antisense, RNA1 (ZFAS1) lncRNA can enhance the Notch-related HES-1 and the Notch intracellular domain (NICD) expression in cells (Gao et al., 2017), thereby promoting the Notch1 upregulation in HNSCC cells in a manner conducive to enhanced metastatic progression (Kolenda et al., 2019).

### NF-κB signaling pathway

In mammalian cells, the NF-κB transcription factor is assembled through the heterodimerization of five different subunit transcription factors: NF-κB/p105, NF-κB/p100, RelA/p65, RelB, and c-Rel (Gupta et al., 2020). These multiple NF-BNF-κB conformations can modulate the target gene expression depending on the situation after dimerization. NF-κB signaling has been shown to play a central role in many human diseases, including neurodegenerative disease, cardiovascular disease, arthritis, and various cancers, including OSCC.

In a variety of disease situations, studies have revealed interactions between NF-κB (Wu et al., 2018) and the transcription factors NKILA42, MALAT142, and HOX transcript antisense RNA (HOTAIR) and the host gene (Özeş et al., 2016) of miR-31 (miR31HG) (Jin et al., 2016) and Gm4419 (Wen et al., 2017). However, relatively few studies have examined these interactions or their clinical significance in the context of OSCC.

The activation of the NF-κB pathway is critical to the EMT induction and maintenance and supports the overall metastatic process. Lower levels of NKILA expression are associated with higher rates of metastasis and poor prognostic outcomes in TSCC patients, which is consistent with its role as a suppressor of tumor growth and with its known capacity to block NF-κB activation by reducing IκBα phosphorylation (Gupta et al., 2020). In one report, Zhou et al. (2015) demonstrated that MALAT1 knockdown suppressed nuclear NF-κB p65, β-catenin, and p-β-catenin accumulation, suggesting that MALAT1 may coordinate signaling between the NF-κB, Wnt, and the Notch pathways to shape the EMT process. Major NF-κB target gene TNF-α is downregulated by the NKILA overexpression, which is consistent with the transcription factor’s capacity to inhibit NF-κB signaling. The siRNA-mediated knockdown of MALAT1, conversely, suppresses OSCC cell NF-κB activation (Zhou et al., 2015). The transmembrane NRP1 glycoprotein is commonly expressed in bladder cancer (Saban et al., 2010), pancreatic cancer (Dallas et al., 2008), and oral cancer.

Moreover, its upregulation is associated with enhanced IκB phosphorylation and nuclear p65 translocation with E-cadherin and β-catenin downregulation and vimentin and N-cadherin upregulation (Raimondi et al., 2016). This suggests that NRP1 may activate NF-κB signaling to induce the EMT induction (Chu et al., 2014). By binding Hsp90, ORAOV1-B can increase its stability and thereby modulate the function of specific client proteins, including IκB, ultimately promoting NF-κB signaling and TNF-α expression, which further enhance NF-κB activation and EMT induction in OSCC (Luo et al., 2021).

## Significance of lncRNAs as regulators of other oral squamous cell carcinoma-associated processes

In addition to the mechanisms and pathways described above, it has been found that several lncRNAs indirectly affect EMT induction and the progression of metastases in OSCC, as shown in Table 2.

**TABLE 2:** LncRNAs interact with miRNAs in OSCC cells to shape the EMT induction.

Function	Author, publication year	LncRNA	miRNA	Protein	References
Promotion of tumor migration	Liu, X., et al., 2018	NEAT1	miR-365	RGS20	Liu et al. (2018b)
	Xu, C.H., et al., 2019	HOXA11-AS	miR-98-5p	YBX2	Niu et al. (2020)
	Li, W., et al., 2020	HOXC13-AS	miR-378g	HOXC13	Li et al. (2020)
	Wang, C., et al., 2021	LINC0664	miR-411-5p	KLF9	Wang et al. (2021a)
	Jiang, X., et al., 2020	LINC00319	miR-199a-5p	FZD4	Jiang et al. (2020)
	Wang X., et al., 2021	ZEB1-AS1	miR-23a	—	Wang et al. (2021b)
	Cao, X.H., et al., 2021	PSMA3-AS1	miR-136-5p	FN1	Cao et al. (2021)
	Qin, H., et al., 2022	LINC01123	miR-34a-5p	—	Qin et al. (2022)
	Wang, Z., et al., 2020	LINC00958	miR-185-5p	YWHAZ	Wang et al. (2020a)
	Xia, Y.C., et al., 2021	TSPEAR-AS2	miR-487a-3p	PPM1A	Xia et al. (2021)
	Kou, N., et al., 2019	H19	HMGA2	Let-7a	Kou et al. (2019)
Inhibition of tumor migration	Zeng, B., et al., 2019	GAS5	miR -21	PTEN	Zeng et al. (2019)
	Tan, J.W., et al., 2019	MEG3	miR-548d-3p	SOCS5/SOCS6	Tan et al. (2019)
	Wang, Q. et al., 2021	SNHG16	miR-17-5p	CCND1	Wang et al. (2021c)

### Function of lncRNAs in regulating the invasion and metastasis of tumor cells

Various lncRNAs have been found to promote metastasis and tumor cell invasion. For instance, the lncRNA AC132217.4 can upregulate the expression of IGF2 to increase OSCC invasion, while Krüppel-like factor 8 (KLF8) regulates the AC132217.4 expression through upstream binding (Li et al., 2017). In HNSCC cells, LINC00460 can promote nuclear PRDX1 translocation, thereby facilitating the EMT activation (Jiang et al., 2019). Alternatively, MIR31HG can promote HNSCC cell progression through the cell cycle and associated proliferation by targeting HIF1A and P21 (Wang et al., 2018b). The HULC (highly upregulated in liver cancer) lncRNA can influence the EMT activity and the expression of other essential proteins such as cyclin D1, MMP-9, Bax, and Bcl-2. These proteins have a key role in tumor cell proliferation and invasion (Su et al., 2019). In OSCC, the PRKG1-AS1 lncRNA also regulates the EMT to shape essential malignant processes such as growth and invasion (Wu et al., 2020). At the same time, the MYOSLID lncRNA modulates a partial EMT program in OSCC cells to promote invasion and metastasis (Xiong et al., 2019). BANCR (BRAF-activated lncRNA) can enhance OSCC cell migration and proliferation by the MAPK pathway (Yao et al., 2021).

Many lncRNAs have been shown to influence the metastatic progression of OSCC through their ability to interact with specific miRNAs or other factors. For example, the NEAT1 (nuclear paraspeckle assembly transcript 1) lncRNA can control invasion and proliferation in OSCC cells *via* the miR-365/RGS20 pathway (Liu et al., 2018b). OSCC cells proliferate more aggressively while HOXA11-AS binds to miR-98-5p and encourages YBX2 (Y-box binding protein 2) overexpression. HOXC13 is upregulated by its antisense lncRNA transcript (HOXC13-AS), which can bind to sequester miR-378g, suppressing OSCC cell malignancy (Li et al., 2020). By acting as a molecular sponge for miR-411-5p, LINC0664 can enhance the expression of KLF9 and associated OSCC cell EMT induction, proliferation, migration, and invasion, whereas KLF9 binds to the LINC00664 promoter region, establishing a LINC00664/miR-411-5p/KLF9 regulatory feedback loop that governs the ability of OSCC tumors to progress (Wang et al., 2021a). Through a LINC00319/miR-199a-5p/FZD4 regulatory axis, the CCL18-induced LINC00319-mediated sequestration of miR-199a-5p promotes angiogenic, proliferative, and metastatic activities in OSCC cells that stimulate the EMT induction (Jiang et al., 2020). In OSCC, the ability of ZEB1-AS1 to bind miR-23a underlines its prooncogenic activity (Wang et al., 2021b), with PSMA3-AS1 sequestering miR-136-5p, and thus promotes the upregulation of the downstream target gene FN1, with the lncRNA thus contributing to more invasive tumor cell growth through the miR-136-5p/FN1 axis (Cao et al., 2021). Due to its capacity to sequester miR-34a-5p, increased LINC01123 expression in OSCC has been associated with poor patient outcomes and increased malignancy at the cellular level (Qin et al., 2022). Higher LINC00958 levels are also present in OSCC tumors wherein they can promote oncogenic processes *via* the miR-185-5p/YWHAZ axis (Wang et al., 2020a). PRM1A is indirectly upregulated by TSPEAR-AS2 *via* its ability to bind and sequester miR-487a-3p during OSCC progression (Xia et al., 2021).

### Functions of lncRNAs as suppressors of tumor metastasis and invasion

The USP17-SNAI1 pathway is regulated by the deubiquitinating enzyme USP17, and LINC02487’s overexpression is sufficient to prevent proliferation, migration, and invasive growth (Feng et al., 2020a). Through its ability to target ROCK1, LOC441178 can constrain squamous carcinoma cell invasion and migration. (Xu et al., 2018). Similarly, TINCR suppresses OSCC tumor growth by regulating JAK2/STAT3 signaling cascades (Zhuang et al., 2020). Malignant cells exhibit high levels of expression of the let-7 miRNA cluster, which is essential as a regulator of the EMT induction and other malignant processes (Shimono et al., 2015). When H19 is silenced, let-7a-mediated HMGA2 expression is impaired, and the HMGA2-mediated EMT process is disrupted (Kou et al., 2019). Furthermore, GAS5 can suppress proliferation, metastasis, invasion, and EMT within OSCC cells through the miR-21/PTEN axis (Zeng et al., 2019). At the same time, MEG3 is a lncRNA that regulates miR-548d-3p/SOCS5/SOCS6 signaling to control the JAK-STAT signaling cascade, ultimately suppressing OSCC cell migration and enhancing apoptotic death (Tan et al., 2019). Functionally, miR-17-5p sequestration and cyclin D1 overexpression are mediated by the SNHG16 lncRNA and can affect OSCC cell malignancy and EMT induction. Cyclin D1 overexpression and miR-17-5p downregulation abolish the prevention of OSCC development caused by the underlined lncRNA (Wang et al., 2021c).

## Future perspectives of LncRNAs in oral squamous cell carcinoma therapy

There is substantial evidence that the EMT plays a crucial role in OSCC’s chemoresistance and disease progression (Chandra Gupta and Nandan Tripathi, 2017), with multiple signaling molecules participating in mutually reinforcing functions in these pathways. Notably, lncRNAs have emerged as crucial regulators of such EMT induction and chemoresistance, contributing to establishing key malignant feedback loops within tumor cells that induce OSCC progression. Cisplatin is generally used as a first-line chemotherapeutic agent to treat OSCC patients. Wang et al. (2020b) determined that MALAT1 can contribute to resistance against this drug, owing to its ability to regulate the EMT induction, regulate the P-glycoprotein expression, and promote the PI3K/AKT/mTOR signaling. The transcription of the lncRNA CYTOR (cytoskeleton regulator RNA) is stimulated by the FOXD1 transcription factor’s interaction with the region upstream of the lncRNA’s promoter. It has the potential to act as a competitive endogenous RNA (ceRNA) that interferes with miR-1252-5p and miR-3148 activity, thereby facilitating LPP upregulation and serving as a considerable mediator of FOXD1-associated EMT induction and chemoresistance in OSCC progression (Meng et al., 2021).

OSCC patients experience high tumor metastasis and recurrence rates, contributing to the high mortality. However, there are currently no biomarkers available that can reliably guide early-stage OSCC diagnosis and treatment. Given the diversity and disease-specific expression patterns of lncRNAs, they demonstrate great promise as targets for diagnosing or treating OSCC. Indeed, several reports have shown that 2+ specific lncRNAs can, when analyzed in combination with tumor biomarkers, enable the more reliable detection of these tumors compared to single biomarker analyses. As such, investigations of lncRNAs and tumor biomarkers offer the opportunity to improve the sensitivity and accuracy of cancer diagnoses (Chen et al., 2021a), protecting patients from the harm associated with misdiagnosis.

Several reports have emphasized the prognostic relevance of lncRNAs in OSCC (Table 3). For example, the upregulation of PSMA3-AS1 is associated with poor patient outcomes (Cao et al., 2021). According to the reported study by Feng et al., the lncRNA SLC16A1-AS1 has the potential to act as a key mediator of OSCC cell proliferation, acting through modulating the cell cycle. The knockdown of SLC16A1-AS1 in SCC25 and CAL27 cell lines contributes to impaired growth and colony-forming activity, and this lncRNA is an attractive prognostic biomarker (Feng et al., 2020b). In another study, the lncRNA CEBPA-AS1 was associated with poor predictive outcomes. It enhanced OSCC progression through its ability to target the CEBPA/Bcl2 axis82. At the same time, LEF-AS1 interacts with LATS1 to suppress Hippo signaling in OSCC cells, indicating that this lncRNA may offer value as a prognostic and therapeutic target against the underlined carcinoma (Zhang et al., 2019c). The FOXD2-AS1 lncRNA is generally upregulated in OSCC patient tissues and correlated with poor predictive outcomes and a poor pathological grade (Liang et al., 2020). According to Tu et al., the intronic regions of the MIR31HG lncRNA were found to contain miR-31. They revealed that there was a correlation between the greater levels of MIR31HG and poor survival in stage-IV OSCC patients.

**TABLE 3:** LncRNAs influence OSCC patient prognostic outcomes.

Author, published year	LncRNA and expression	References
Cao, X., et al., 2021	PSMA3-AS1↑	Cao et al. (2021)
Feng, H., et al., 2020	SLC16A1-AS1↑	Feng et al. (2020b)
Guo, Y., et al., 2018	CEBPA-AS1↑	Guo et al. (2018)
Zhang, c., et al., 2019	LEF1-AS1↑	Zhang et al. (2019c)
Liang, X., et al., 2020	FOXD2-AS1↑	Liang et al. (2020)
Tu, H.F., et al., 2022	MIR31HG↑	Tu et al. (2022)
Yu, Y., et al., 2021	HOTAIRM1↑	Yu et al. (2021)
Yuan, S.J., et al., 2021	LINC01793↑	Yuan et al. (2021)
Zhen, X., et al., 2020	SAMMSON↑	Zheng et al. (2020)
Lee, E.Y., et al., 2021	lncRNA H19 (Hypomethylation)↑	Lee et al. (2021)
Zhao, C., et al., 2018	AC013268.5↑, RP11.65 L3.4↑, RP11.15A1.7↓	Zhao et al. (2018)
Diao, P.F., et al., 2019	RP11-366H4.1↑, LINC01123↑, RP11-110I1.14↑, CTD-2506J14.1↓	Diao et al. (2019b)
Chen, Y., et al., 2021	AL139158.2↓, AL031985.3↓, AC104794.2↓, AC099343.3↑, AL357519.1↑, SBDSP1↑, AC108010.1↓	Chen et al. (2021b)
Huang, G.Z., et al., 2019	AFAP1-AS1↑, AQP4-AS1↓, C11orf97↓, HOTTIP↑, LINC00460↑, LINC01234↑, SLC8A1-AS1↓	Huang et al. (2019)
Jiang, Q., et al., 2021	PTCSC2↓, AC099850.3↑, LINC01963↓, RTCA-AS1↓, AP002884.1↑, UBAC2-AS1↑, AL512274.1↓, MIR600HG↑, AL354733.3↓	Jiang et al. (2021)
Li, T., et al., 2022	STARD4-AS1↓, AC099850.3↑, AC090246.1↑, ALMS1-IT1↑, AC021087.4↑, MIAT↓, HOTAIRM1↑, AL512274.1↓	Li et al. (2022)
Miao, T.T., et al., 2020	LINC01629↑, AC083967.1↑, AC067863.1↑, AC022092.1↑, AC005532.1↑, BX323046.1↓, PRR29-AS1↑	Miao et al. (2020)
Wu, L., et al., 2022	DDN-AS1↑, AL035458.2↑, LINC01281↓, AC245041.2↑	Wu et al. (2022)
Xu, Z., et al., 2022	AFAP1-AS1↑, ALMS1-IT1↓, HLA-F-AS1↓, KANSL1-AS1↓, LINC-PINT↑, LINC00567↓, LINC00689↓, LINC00877↓, LINC00958↑, LINC01191↓, LINC01281↓, NPSR1-AS1↑, PRKG1-AS1↑, WDFY3-AS2↑	Xu et al. (2022)
Yang, Q., et al., 2022	AC079684.2↑, AC092115.4↑, LINC01644↑, LINC01410↑, AL355574.1↑, AC091271.1↓, LINC00630↑, ALMS1-IT1↑, LINC00992↓, AC099850.4↑, AC005288.1↑, AC107027.3↓, JPX↑, LINC01775↑ and PTOV1-AS1↑	Yang et al. (2022)

In contrast, higher miR-31 levels predicted nodal metastasis among stage I–III patients (Tu et al., 2022). A close association between HOTAIRM1 and OSCC tumor development has been reported, with this lncRNA serving as a cell cycle regulator and candidate prognostic/therapeutic target in OSCC (Yu et al., 2021). Higher LINC01793 levels are associated with a poorer OSCC prognosis for patients and poor clinicopathological features, showing that this lncRNA is an oncogenic mediator in cancer (Yuan et al., 2021). Serum Samson levels exhibit a high diagnostic sensitivity and specificity relative to other serum biomarkers (i.e., CEA, SCCA, and TSGF), indicating that this lncRNA may be an essential regulator of progression in OSCC to serve as a diagnostic and prognostic biomarker (Zheng et al., 2020). Moreover, an elevated level of H19 lncRNA expression is evident in OSCC cells. Compared to healthy control cells, this overexpression frequently coexists with H19 promoter hypomethylation, increasing the possibility that this hypomethylation could serve as a candidate of oral cancer prognostic biomarker (Lee et al., 2021).

Prognostic lncRNA profiles for OSCC cancer have been revealed by bioinformatics analyses in a substantial way (Table 3). Based on the Zhao et al. (2018) study, a panel of three lncRNAs was associated with OSCC patient prognosis. Similarly, Diao et al. (2019b) developed a risk score model based on four lncRNAs that has predictive implications for several clinicopathological parameters. Chen et al. (2021b) identified seven lncRNAs that together make up an immune-related lncRNA prognostic signature (IRLPS) capable of classifying patients with HNSCC into IRLPS-high and -low subgroups by analyzing their associations with immune function. In this view, these predictive methods could distinguish between the distinct tumor microenvironmental characteristics of OSCC patients. Moreover, Huang et al. (2019) detected seven prognosis-associated lncRNAs, while Jiang et al. (2021) defined a prognostic marker for OSCC patients consisting of autophagy-associated lncRNAs. Li et al. (2022) developed a prediction model using eight ferroptosis-associated lncRNAs and found that while high-risk patients had greater levels of AC099850.3, AC090246.1, ALMS1-IT1, AC021087.4, and HOTARM1, low-risk patients had higher levels of STARD4-AS1, MIAT, and AL512274.1 expression. Miao et al. (2020) identified seven lncRNAs with potential clinical and prognostic relevance in OSCC. Wu et al. (2022) reported a prognostic signature consisting of four glycolysis-associated lncRNAs capable of differentiating between low- and high-risk patients. Xu et al. (2022) found that a signature composed of 14 lncRNAs was able to predict OSCC patient prognosis and offer insight regarding immune cell status that may support immunotherapeutic intervention efforts. Yang et al. (2022) found several m6A-associated lncRNAs that could be used to predict the outcome of OSCC, and they used the underlined lncRNAs to make a prediction model.

The TME is a primary focus of recent cancer-related research, consisting of the stromal cells, immune cells, and extracellular matrix (ECM) proteins that form the local tumor niche (Quail and Joyce, 2013). The TME in OSCC tumors is typically hypoxic and has immunosuppressive and proinflammatory properties. The complex biomolecular signaling processes within the TME ultimately shape oncogenesis, metastatic progression, and treatment-related outcomes (Tang et al., 2021). Hypoxia can promote enhanced EMT induction in epithelial-like OSCC cells (Huang et al., 2018b). In patients with high-grade invasive OSCC, programmed-death 1 (PD-1) receptor/programmed-death ligand (PD-L1) overexpression has been seen in macrophages and dendritic cells. Similar findings have been observed for mesenchymal OSCC cells *in vitro* (Hirai et al., 2017; Wu et al., 2021). Tumor-associated macrophages (TAMs) with an M1 phenotype can drive EMT progression and promote cancer-stem cell (CSC) development in OSCC through the upregulation of MMP-14 and MME (You et al., 2022). In OSCC, stromal CAFs express high levels of lysyl oxidase (LOX), which promotes matrix collagen remodeling and stiffens the matrix in a way that promotes OSCC progression *via* the FAK phosphorylation pathway (Zhang et al., 2021). Hu et al. (2022) determined that neutrophils can control EMT and JAK2/STAT3 signaling in OSCC *via* chemerin, thus promoting progression. Interactions between the malignant cells and the TME are essential for EMT induction and multidrug resistance (MDR) onset (Erin et al., 2020). EMT induction consumes large amounts of energy, compromises T cell activation, and contributes to immune evasion (Bai et al., 2021).

The development of new therapeutic regimens that are more effective and maintain individualized treatment prognostic evaluation and diagnosis can be greatly helped by extensive studies to reveal the molecular mechanism underlying the role of specific lncRNAs in the context of tumorigenesis, metastatic progression, and production functions. Several clinical trials have explored the use of lncRNA-targeting therapeutics such as siRNAs and antisense oligonucleotides. These therapeutic modalities are particularly promising given that many lncRNAs exhibit tissue- or tumor-specific expression patterns that make them superior pharmacological targets, given that traditional small molecule inhibitor drugs show low target protein specificity levels. In this view, the lncRNAs have considerable potential as candidate clinical biomarkers and therapeutic targets. However, they will need to be tested in more clinical trials to make sure they are important to patient outcomes before they can be used more widely in clinics.
